# Stroke thrombolysis in a middle-income country: A case study exploring the determinants of its implementation

**DOI:** 10.3389/fneur.2022.1048807

**Published:** 2022-11-24

**Authors:** Wen Yea Hwong, Sock Wen Ng, Seng Fah Tong, Norazida Ab Rahman, Wan Chung Law, Zurainah Kaman, Sing Keat Wong, Santhi Datuk Puvanarajah, Sheamini Sivasampu

**Affiliations:** ^1^Institute for Clinical Research, National Institutes of Health, Ministry of Health Malaysia, Selangor, Malaysia; ^2^Julius Center for Health Sciences and Primary Care, University Medical Center Utrecht, Utrecht University, Utrecht, Netherlands; ^3^Department of Family Medicine, Universiti Kebangsaan Malaysia, Selangor, Malaysia; ^4^Neurology Unit, Department of Medicine, Sarawak General Hospital, Ministry of Health Malaysia, Kuching, Malaysia; ^5^Department of Neurology, Hospital Kuala Lumpur, Ministry of Health Malaysia, Kuala Lumpur, Malaysia

**Keywords:** acute stroke care, intravenous thrombolysis, developing countries, translational research, facilitator, barrier

## Abstract

**Introduction:**

Translation of evidence into clinical practice for use of intravenous thrombolysis in acute stroke care has been slow, especially across low- and middle-income countries. In Malaysia where the average national uptake was poor among the public hospitals in 2018, one hospital intriguingly showed comparable thrombolysis rates to high-income countries. This study aimed to explore and provide in-depth understanding of factors and explanations for the high rates of intravenous stroke thrombolysis in this hospital.

**Methods:**

This single case study sourced data using a multimethod approach: (1) semi-structured in-depth interviews and focus group discussions, (2) surveys, and (3) review of medical records. The Tailored Implementation of Chronic Diseases (TICD) framework was used as a guide to understand the determinants of implementation. Twenty-nine participants comprising the Hospital Director, neurologists, emergency physicians, radiologists, pharmacists, nurses and medical assistants (MAs) were included. Thematic analyses were conducted inductively before triangulated with quantitative analyses and document reviews.

**Results:**

Favorable factors contributing to the uptake included: (1) cohesiveness of team members which comprised of positive interprofessional team dynamics, shared personal beliefs and values, and passionate leadership, and (2) facilitative work process through simplification of workflow and understanding the rationale of the sense of urgency. Patient factors was a limiting factor. Almost two third of ischemic stroke patients arrived at the hospital outside the therapeutic window time, attributing patients' delayed presentation as a main barrier to the uptake of intravenous stroke thrombolysis. One other barrier was the availability of resources, although this was innovatively optimized to minimize its impact on the uptake of the therapy. As such, potential in-hospital delays accounted for only 3.8% of patients who missed the opportunity to receive thrombolysis.

**Conclusions:**

Despite the ongoing challenges, the success in implementing intravenous stroke thrombolysis as standard of care was attributed to the cohesiveness of team members and having facilitative work processes. For countries of similar settings, plans to improve the uptake of intravenous stroke thrombolysis should consider the inclusion of interventions targeting on these modifiable factors.

## Introduction

Effective management in the early stages of an acute ischemic stroke is crucial to reduce mortality and morbidity. Recent advancement in stroke treatment recommends the use of intravenous thrombolysis within 4.5 h of an acute ischemic stroke (1).

Nevertheless, translation of this evidence into clinical practice remains challenging. In developed countries, the rate of intravenous thrombolysis among patients who presented with acute ischemic stroke ranged from 13.7% in the United States in 2018 (2), 11.7% in the United Kingdom to 20.6% in the Netherlands, both in 2017 (3). The discrepancy between clinical guidelines and actual clinical practice was more apparent among low- and middle-income countries with an average of 3% uptake of the therapy (4).

The extent of success in its adoption were contributed by multiple factors. From the perspective of healthcare providers, lack of training and self-confidence to administer therapy, poor communication, limited resources and incentives were reported as key barriers (5, 6). Guideline awareness, work pride and motivation and regular feedback were identified to facilitate the implementation of stroke thrombolysis (6, 7). A majority of these existing studies however, were performed in high-income countries. Challenges in providing intravenous stroke thrombolysis in resource-poor countries can be significantly different and should be acknowledged, particularly when the burden of stroke is higher in these countries. In Ghana, distinctive factors from the high-income countries were found which included the role of sociocultural beliefs and the lack of coverage for acute care in their national health insurance (8). Furthermore, the importance of understanding barriers and facilitators in the implementation of intravenous stroke thrombolysis in a low- and middle-income country was highlighted in studies assessing the effectiveness of different interventions developed to improve the therapy. These interventions were found to produce similar effects, despite their targets on different aspects of stroke thrombolysis. Given the degree of variability between studies, it has been recommended for selection of intervention to address specific challenges in the given context until better evidence emerges (9).

Malaysia is one of the low- and middle-income countries where local regulatory authorities have approved the use of recombinant tissue plasminogen activator (r-TPA) for acute ischemic stroke (10). Nevertheless, the reported national uptake of intravenous stroke thrombolysis among public hospitals in the country has been poor at 1.6% in 2018 (unpublished data: Hiew FL. Stroke Thrombolysis Survey in Ministry of Health Malaysia. 2019). Intriguingly, one public hospital reported comparable rates of the therapy to high-income countries (11). This study therefore, was set to explore and provide an in-depth understanding of factors and explanations for the high rates of intravenous stroke thrombolysis in this hospital, which could explain its differences in comparison to other hospitals in similar socioeconomic settings.

## Methods

### Study design

This study adopted a case study methodology. Qualitative case study allows an in-depth understanding and exploration of the phenomenon of interest within the real-world context (12). In this study, this refers to the exploration of reasons for the high rate of thrombolysis. This case study involved a Ministry of Health hospital which is referred as “Hospital Z.” It is a 1,057-bedded tertiary referral center. Neurology unit is placed under the Medical Department where out of 200 beds, an area of 6-beds was established as an acute stroke unit. A weighing bed is reserved for patients indicated for thrombolysis.

### Research team

The research team comprised of 4 women and 2 men. SFT is a family medicine specialist with experience in qualitative research. WYH, SWN and NAR are trained in qualitative research, with medical and pharmacy backgrounds, and have been conducting clinically-related stroke research. WYH and NAR hold higher qualifications in epidemiology. SS is a public health specialist who heads health systems research. WCL is a neurologist with specific interest in stroke care.

Researchers have no prior relationships with the participants, except for WCL who has worked directly or indirectly with participants for patient management. WCL was not involved in data collection. His roles were to provide expert opinions to make sense of the data. It was made clear to the participants that the interviews were conducted to understand their experiences in implementing thrombolysis.

### Tailored Implementation in Chronic Disease framework

The TICD is an implementation framework which guides understandings on the determinants of implementation change in clinical practice and of recent, in stroke care. There were 57 determinants of practice in seven domains which include factors related to individual health professionals, professional interactions, guidelines, incentives and resources, patients, capacity for organizational change, and social, political and legal (13). As this framework provides an overview of the common barriers and facilitators for programme implementation in clinical practice, it was used as a guide to develop interview guides for data collection. Instinctively, determinants from the framework were also applied when we conducted the initial line by line coding during data analysis.

### Data collection

#### In-depth interviews and focus group discussions

Semi-structured interviews were conducted to provide data on participants' thoughts and work processes that lead to the decision for thrombolysis. Interview guides for each profession were developed based on literature (8, 13, 14), expert opinions, and guided by the TICD framework. The interview guides were pilot-tested among medical professionals outside our study sites and adapted following their feedback (Supplementary material 1).

A purposive sampling was conducted among healthcare providers who were directly involved in providing the therapy and senior administrators who were authorized to make decisions. We included healthcare providers with at least 6-months experience in the study site to augment the validity of their shared experience as a reflection of the actual situation. Healthcare providers were chosen from a variety of profession and therefore, with rather different but at times, overlapping roles in the provision of intravenous stroke thrombolysis. As sample size for qualitative research is dependent on the saturation of information necessary to answer the study objectives, we conducted at least one interview for each group of healthcare providers. A reported average number of interviews needed to reach saturation is between 9 and 17 (15).

To facilitate recruitment, phone calls were made to the Heads of Department of each specialty involved. Potential participants were recommended and engagements were made *via* text messages and telephone calls. Thirty-two participants were invited to participate and none declined. However, 3 participants could not attend the interview because of hospital admission (*n* = 1) and emergency calls (*n* = 2). Table 1 shows the distribution of participants, by their professions and the types of interview conducted.

**Table 1:** Distribution of participants by profession and types of interview conducted.

**Participants**	**Number** **of** **participants**	**Types of interview**
Neurologists	2	In-depth interview
ED physician and medical officer	2	In-depth interview
Radiologist	1	In-depth interview
Medical Department HOD and medical officers	3	In-depth interview
Hospital director	1	In-depth interview
Radiographers	4	Focus group discussion
ED nurses	4	Focus group discussion
Neurology unit nurses	6	Focus group discussion
ED medical assistants (MAs)	4	Focus group discussion
Pharmacists	2	Focus group discussion
Total	29	

ED, emergency department; HOD, Head of department.

Four researchers (WYH, SWN, SFT, NAR) took turns to conduct the interviews. There were nine IDIs and five FGDs. None of the interviews were repeated. The main language used was English except for the FGDs with the nurses, medical assistants (MAs) and radiographers, which interviews were conducted in both English and Malay languages. In many healthcare systems, the roles of ground staff including nurses, MAs and radiographers are often considered less important in comparison to clinicians (16). Typically, this steep hierarchical gradient results in the fear to voice out their opinions especially on negatively-related issues. The conduct of FGDs is aimed to encourage staff participation and provision of feedback comfortably in the presence of their peers. Additionally in FGDs, the role of moderators would likely to be perceived as less domineering than interviewers in an IDI. Due to restrictions following COVID-19 pandemic, these interviews were conducted virtually using a video conferencing platform. Each interview lasted about 50 min. Interviews were recorded visually for the purpose of transcribing and to observe non-verbal cues. All records were subsequently transcribed with clear verbatim. Transcripts were not returned to participants but were rechecked randomly to ensure that the contents matched the audio recording.

#### Surveys and medical record reviews

Quantitative surveys and medical chart reviews were conducted to supplement findings from the qualitative data by evaluating available resources and quantifying reasons for not receiving thrombolysis. For surveys, information on hospital facilities, number of staff, rates of thrombolysis among ischemic stroke patients, and services available were collected in a predetermined data collection sheet. The review of medical records involved obtaining a list of ischemic stroke patients admitted between June and December 2019 from the hospital's stroke registry and having their medical records reviewed to investigate reasons for not receiving thrombolysis. A systematic sampling was conducted by including every 5th patient with a minimum of 15 patients every month. In total, 105 patient records were included.

### Data analysis

Data from different sources were analyzed separately before being compiled for cross examination and triangulation.

First, the transcripts were de-identified and compiled using Nvivo 12 software for data management (17). Second, line-by-line coding was performed by WYH and SWN independently using TICD framework as an initial guide to provide a bearing on possible determinants to focus on. During the coding exercise, the codes were not restricted to the categories available within TICD. Instead, codes were also generated inductively based on understanding and in-depth analysis from the transcripts. To ensure congruence, the codes were subsequently compared and discrepancies were resolved in a discussion. Each code was also distinguished by the participants' profession to understand its context and to seek for patterns across the codes. The codes were then categorized into bigger constructs and sorted into domains.

The third step involved analysis of findings from the surveys and medical record reviews descriptively. Next, triangulation of data was performed. This entailed several discussions among the research team on presentation of the main findings based on coded data, transcripts and how the quantitative results supplemented those findings. During these discussions, we also discussed the choice of codes and its transcripts to be presented by looking through different transcripts to ensure that the message quoted is clear, direct and independent. Finally, feedback from content experts and peers who were not directly involved in the data analysis were obtained to aid making sense of the data.

## Results

### Stroke care services in Hospital Z

Management of stroke cases is handled by the stroke team led by two neurologists. This responsibility is shared across the Emergency, Radiology and Medical Departments. Intravenous stroke thrombolysis has been available since 2013 during office hours but from 2015, the therapy has been expanded to 24 h daily. The number of ischemic stroke admissions increased from 116 patients in 2013 to 610 in 2019. Likewise, the rate of intravenous thrombolysis was about 5% in 2013 and 2014 before it rose to a range between 11.1 and 20.8% in the later years (Table 2).

**Table 2:** Rate of intravenous stroke thrombolysis in Hospital Z from 2013 to 2019^a^.

**Year**	**Total admission** **for ischemic** **stroke patients**	**Number of stroke** **activation calls**	**Number of** **patients** **thrombolyzed**	**Rate of** **thrombolysis (%)**	**Rate of true** **positive activation** **calls^b^ (%)**
2013	116	na	6	5.2	na
2014	249	na	12	4.8	na
2015	368	59	41	11.1	69.5
2016	440	115	62	14.1	53.9
2017	553	124	87	15.7	70.2
2018	568	148	118	20.8	79.7
2019	610	na	92	15.1	na

^a^Sourced from data collection for stroke registry in Neurology Unit in Hospital Z;

b true positive activation calls refer to stroke activation calls which ended up with thrombolysis being conducted; na: not available.

Admission of acute stroke patients goes through ED. Patients who present with acute neurological deficit during triage assessment would undergo a fast-tracked standardized workflow which includes ED medical officer assessment and a Computed tomography (CT) request before a stroke activation call is prompted. Computed tomography or CT angiography imaging facilities are not available in ED and patients are sent to the Radiology Department located in a different building. Computed tomography imaging facilities are available for 24 h. Although magnetic resonance imaging (MRI) or MR angiography are only available during office hours, its use for acute stroke cases can be requested as necessary.

A dedicated stroke team comprising a neurologist, a medical officer, and a nurse would attend to the patients during office hours. After office hours, the stroke nurse or a medical staff nurse is on standby while the neurologists are available for remote consultations. Once patients are identified to be eligible for thrombolysis, they will be consented and sent to the acute stroke unit. No allocated elevators are available for patient transport.

### Factors influencing the uptake of intravenous stroke thrombolysis

**Figure 1:**
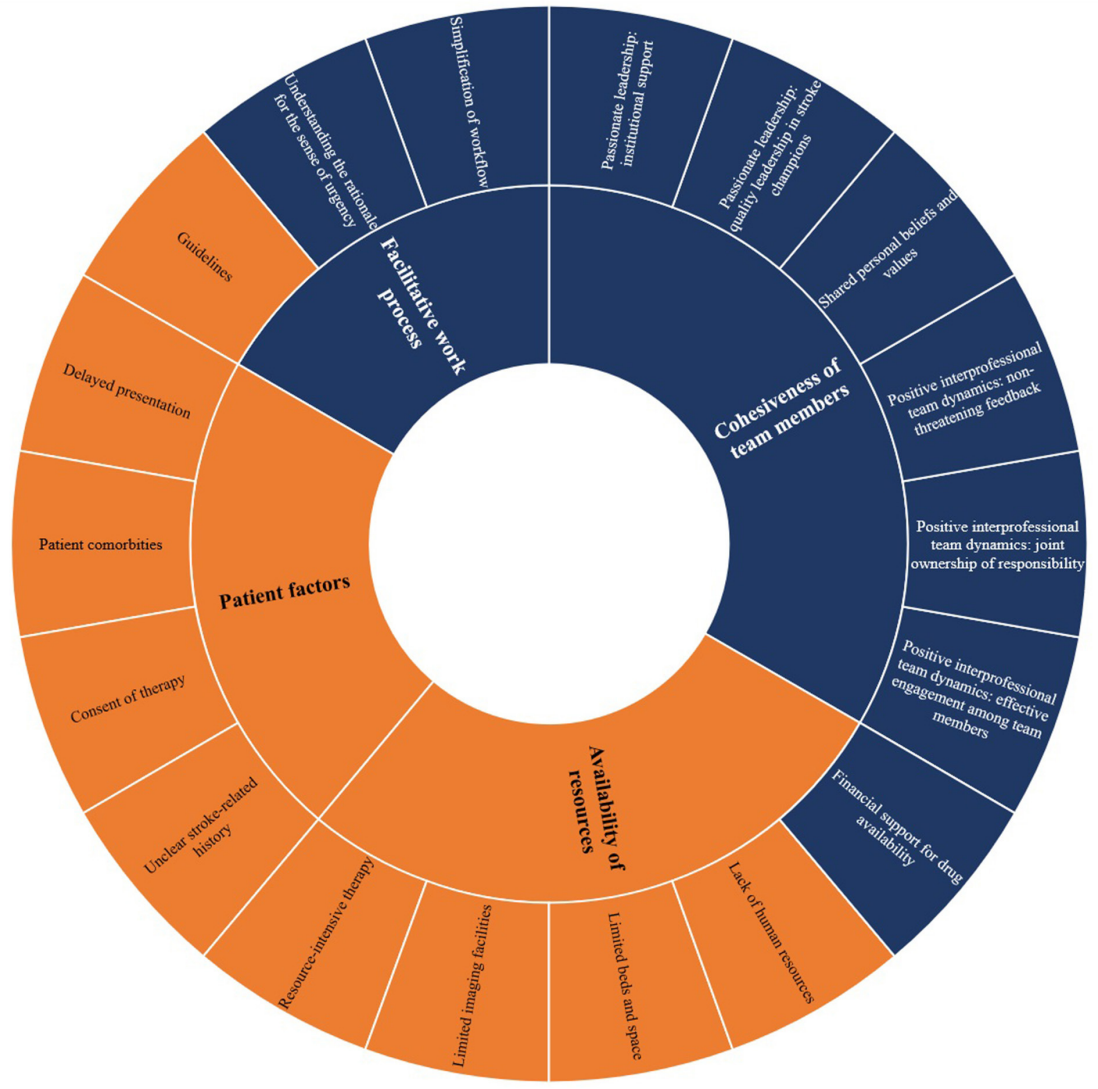
Factors influencing the uptake of intravenous stroke thrombolysis in Hospital Z. Sunburst diagram reflecting each domain with its respective constructs. Colors refer to facilitators (blue) and barriers (orange).

**Figure 2:**
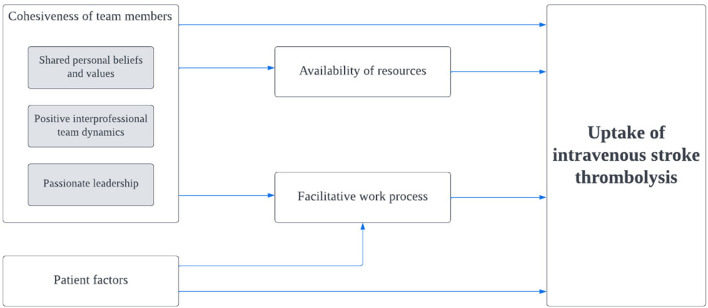
Relationship between contributing factors and the uptake of intravenous stroke thrombolysis. Gray boxes refer to constructs within the specified domain.

The rates of thrombolysis in Hospital Z could be attributed and explained from four main factors discussed below (Figure 1; Supplementary material 2). Figure 2 shows the relationship of the contributing factors to the uptake of thrombolysis. Cohesiveness of team members and facilitative work process were found to ease the service provision. Patient factors and availability of resources impeded the uptake of this therapy although the latter was innovatively optimized.

#### Cohesiveness of team members

##### Positive interprofessional team dynamics

###### Effective engagement among team members

Having effective communication and willingness to engage with team members were key facilitators to achieve effective dynamics within an interdisciplinary team. The neurology team played a significant and engaging role in building rapport and initiating interdisciplinary meetings to facilitate better communication between departments. “*I spent a lot of time going down to the radiology department, talking to the radiologist and making myself known to them* (ID 07)*.”* Similar reflections were provided by participants from other departments: “*She (neurologist) came to ED to discuss with our Head of Department regarding stroke protocol (ID 04)”* and “*Our hospital became more active (in the service provision) since Dr X came. He approached us when he wanted to do this (thrombolysis) and asked us to help facilitate (ID 11)”*.

Besides, team members from different professions were interdependent and supportive of each other. One MA reflected (ID 06-01): “*Upon stroke activation, sometimes the stroke team or the ED houseman will push the patients for CT scan. They do not rely on MAs, nurses or porters.”* Team members were also approachable. Said one doctor (ID 07): “*Generally, everyone has been somewhat approachable. I think that certainly help in getting and pulling everyone to work together as a team.”*

Such culture has benefitted the sustenance of the therapy beyond Hospital Z's boundaries. For example, thrombolysis has been expanded to district hospitals without neurosurgeons in the state with commitments from the neurosurgical department in Hospital Z to back these hospitals up should complications occur.

###### Joint ownership of responsibility

The joint ownership was demonstrated through trust and confidence in sharing responsibilities. Neurologists trusted the clinical assessments of their medical officers in identifying eligible patients for thrombolysis. Likewise, there was mutual trust between the radiologists and neurologists because the ultimate responsibility to patient care was shared. The neurologists were willing to interpret CT images for stroke patients: “*We tell them (radiologists) that we (are) going to see the scan. They don't have to come and report* (ID 01)*.”* Importantly, the radiologists were comfortable for the task to be handled by the neurologists: “*We are okay with them (neurologists) interpreting the scan. For them (the neurologists) (they need) to have immediate (report), because (they) need to act on the scan finding* (ID 11)*.”*

Besides trust, a positive attitude toward joint sharing of workload from thrombolysis resonated among the participants. Neither did they feel that having thrombolysis has added to their workload nor did they require financial incentives. As noted by one radiographer (ID 08-04): “*I am not burdened because it is indeed our job”* and one nurse said (ID 02-02): “*We only claim off hours. We do not claim (money).”*

Initiatives to cope with constraints of human resources with the aim to improve the quality of stroke care were also seen as a form of joint ownership of responsibility. One doctor explained how they empowered and privileged medical physicians and medical officers to handle the therapy (ID 07): “*At the moment, we don't have trainees. So, a lot of our bouts (are) being covered by (medical) physicians (and) medical officers. When they receive stroke activations, they are the first to attend to patient and when we said “yes” for thrombolysis, they'll be the one(s) helping to push up patient to the ward, administering thrombolysis and monitoring.”* The nurses also had a systematic rotation to support the stroke team: “*So far, there are seven of us. Every month, our nursing sister will arrange the schedule for two nurses (to be on call for stroke activation calls)* (ID 02-06)”.

Nevertheless, presence of mixed opinions on sharing the responsibility to decide for thrombolysis could potentially threaten this dynamic. Emphasizing the hesitancy of the emergency physicians, one doctor (ID 04) said: “*Most of us are not there yet. To say that we would take over the decision to thrombolyze, not all of us are used to it.”* Furthermore, increase in the number of medical subspecialties has led to issues of patient segregation: “*The moment they go into specialized area (to manage patients from) the general medicine side (would be) like: this is not mine. This is somebody else's* (ID 13)”.

###### Non-threatening feedback

A majority of the participants agreed on the importance of having an avenue to provide feedback within and across departments: “*Normally if there is feedback, we will share it in our WhatsApp group. But if there is something confidential that requires face-to-face discussion, we will talk to the staff involved* (ID 06-03)”.

Furthermore, feedback was transferred to ground staff although the initial communications only involved higher authorities. Interestingly, having received negative feedback was accepted as a measure of encouragement: “*They (the neurologists) get upset when it's (stroke is) missed sometimes but not overly upset. Appropriately upset. So, we try not to miss. So, the culture is such that we want to do it well* (ID 09).” More importantly, the participants confided on how their team members were often non-judgemental in handling mistakes occurring at work. Said one MA (ID 06-04): “*If he or she (other MA) happens to make a mistake, we will try to resolve it together.”* One doctor also recalled (ID 09): “*When we call them (the neurologists), they will come and assess. So, it gives us confidence that we wouldn't be blamed”*.

##### Shared personal beliefs and values

Having the intentions and motivations to optimize the therapy were key values portrayed. One doctor shared (ID 01): “*We can achieve what I believe as universal access of acute thrombolysis across the country. I always believe I wanted to do for others what I want them to do for me. Thus, I will try my level best to treat them. I think that's the main drive.”* Echoed by another (ID 07): “*With the introduction of treatments, you can actually help patient(s) to live independent life. That's actually a good motivation for me personally, because you know that your work makes a difference”*.

One nurse reflected how her experiences in the use of thrombolysis influenced her beliefs on the treatment outcomes (ID 02-03): “*There are some patients with power 0 who can (improve) to 3. That makes us satisfied. The thrombolysis seems successful.”* A doctor also shared his confidence on the low bleeding risk from thrombolysis (ID 01): “*From my own experience, hemorrhagic transformation (that) requires neurosurgical intervention (is) not very common. We (are) talking about* <*3%. The benefit outweighs the risk. We're talking about 30% of our stroke patients can become normal or near-normal again. That's the minimum”*.

##### Passionate leadership

###### Quality leadership in stroke champions

The importance of having passionate stroke champions to drive the therapy was reiterated. One doctor said (ID 11): “*I think the person doing it is very important, like—Dr X. I can see his dedication. I think we can all share the enthusiasm.”* Ground staff of Hospital Z also shared their views where they appreciated the working personality of the stroke champions: “*Our bosses are quite hands-on. They don't just give order; they will attend even though they are consultants. That gives us confidence* (ID 09).” Besides that, the nature of them sharing the achievements earned from thrombolysis have provided motivations to the staff: “*Dr X does share with us some awards that they achieved. (This brings) some positive reinforcements for the radiographers. They are the ones who do 24 h shift to scan the patients* (ID 11)”.

###### Institutional support

One doctor reflected on the attitude of the higher authorities and other departments in the hospital toward thrombolysis (ID 01): “*Our radiologists, our hospital director and our head (of) department are very supportive”*.

#### Facilitative work process

##### Simplification of workflow

Difficulties in identifying stroke cases during triaging were attributed to patients with atypical symptoms. “*Patients present with very vague symptoms. The worst would be if the patient keeps on having dizziness but you cannot pinpoint whether there is any obvious neurology deficit or not* (ID 10)”.

To address this, a simplified criteria to assess thrombolysis eligibility was established. As explained by one doctor (ID 01): “*We change the (triaging) protocol to acute neurology deficit. We're very lenient for them to activate and call us.”* Table 2 showed an initial dip in the percentage of patients who received thrombolysis after a stroke activation call between 2015 and 2016 but subsequently, a sharp increase from 53.9% in 2016 to 79.7% in 2018.

Besides, patients with acute neurology deficits would be fast tracked for stroke activation and CT imaging. Said one doctor (ID 01): “*We default registration for admission, we'll get consent on the way to CT.”* Workflow for referrals from district hospitals was also simplified where ambulances from district hospitals without CT imaging were encouraged to bypass their own hospital and send suspected stroke patients to Hospital Z directly. As a result of this streamlining process, healthcare providers found it easy to be familiar with their roles. As explained by one doctor (ID 04): “*Overtime, we get comfortable with it that it becomes a reflex. You say that this is stroke, people would know what to do”*.

**Figure 3:**
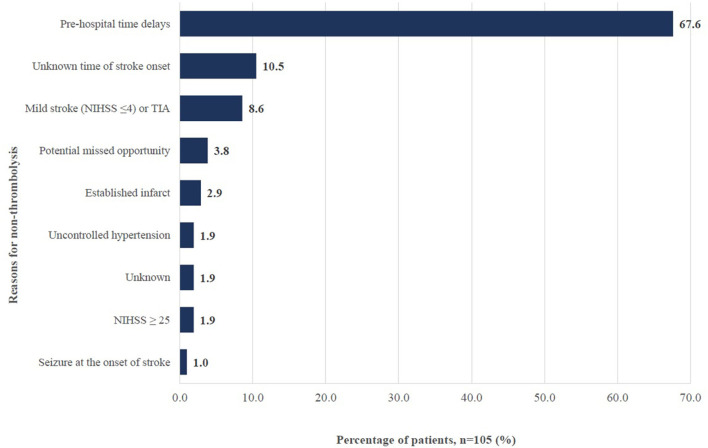
Reasons for non-thrombolysis among ischemic stroke patients in Hospital Z between June to December 2019. TIA: Transient ischemic attack; NIHSS: National Institutes of Health Stroke Scale; potential missed opportunity includes those with delayed referral to CT imaging and delayed assessment by stroke team.

Parallelly from the medical records review, potential in-hospital delays related to workflows accounted only for 3.8% of patients who missed the opportunity to receive thrombolysis due to a delay in referral for CT imaging (*n* = 2) and a delay in assessment by stroke team (*n* = 2), one of which suffered stroke whilst being an inpatient (Figure 3).

##### Understanding the rationale for the sense of urgency

It was coherently agreed that stroke cases should be given a priority. One doctor explained (ID 04): “*Once we suspect that it is stroke, we quickly determine that it is acute and within time. We will quickly activate the stroke thrombolysis.”* The same sense of urgency for stroke patients was shared in radiology department: “*Whenever you say thrombolysis, the radiographers know that we have to stop our elective case(s) and scan the case first* (ID 11)”.

This sense of urgency has been instilled with informal training in the form of briefings, orientations and tagging to senior staff. One doctor highlighted the importance of educating the staff on why stroke cases should be made a priority (ID 11): “*They have to understand why their workflow must be disrupted. I think the point is to make them understand the time constraint. Once they understand that, I think they are more acceptable. Rather than you say, “Thrombolysis, you must scan now.” They don't know why”*.

#### Patient factors

##### Delayed presentation

One suggested reason for delayed presentation was the low public awareness on stroke symptom recognition. One doctor noted (ID 12): “*Once they (patients) have weakness on one side of the body, they will go for massage. When they come, it's already 2 or 3 days (after).”* The public were also unaware of the availability of a time-dependent therapy for stroke: “*(People are) not exposed (to it). Only if they go to the hospital, then would they know about this service* (ID 02-01).” Furthermore, logistic issues, in particular among patients who required hospital transfers due to the lack of CT machines in district hospitals were brought up. Findings from the medical records review supported the importance of this barrier. A total of 67.6% of ischemic stroke patients arrived at the hospital outside the therapeutic window time (Figure 3).

##### Patient comorbidities

Having comorbidities or a severe stroke may contraindicate patients from the therapy. Figure 3 showed that uncontrolled blood pressure levels and seizure upon onset of stroke constituted 2.9% of the reasons for non- thrombolysis whereas 4.8% had either established infarcts or NIHSS score which was ≥25. Patients with poor condition upon arrival could also have their CT imaging delayed, potentially excluding them from the opportunity to be thrombolyzed.

##### Consent of therapy

The issue of consent largely existed due to influence from family members. One doctor argued that it depended on how the risk benefit explanation was provided (ID 14): “*I have seen thrombolysis refusals, maybe one in fifty? Not very common. Because it depends on how you explain to them. If you tell the family member, we give that (thrombolysis) there's a chance you will improve and if we don't, most likely you'll remain like this. Most of the time, they are quite receptive even though you tell them there is a risk of bleeding”*.

##### Unclear stroke-related history

Unclear patient history and language barrier complicated the triaging assessment and subsequently delayed referrals. Explained by a doctor (ID 07): “*Sometimes the problem (is that) patients and family themselves are not forthcoming with regards to their time of onset of stroke. So, we can't really decide on exactly when was (the actual) onset because the history was so unreliable.”* Likewise, this was evident from Figure 3 where 10.5% of ischemic stroke patients did not receive thrombolysis because the onset of their stroke was unknown.

#### Availability of resources

Intravenous stroke thrombolysis is known to be a resource-intensive therapy (ID 01): “*Thrombolysis is labor intensive and we have to respond very quickly. It's every 15 min monitoring. It's costly. We need CT scan. We used to have to call radiologist(s) to get the permission, then we get patient consent for CT and then push their way to CT scan room which is always not next to ED. And then have to interpret CT. There's always a reservation among neurologists in Malaysia because it is labor intensive.”* Working around challenges related to resources thus, had not been easy.

##### Limited imaging facilities

Due to the lack of a CT machine in ED, more time were required to transfer patients to undergo CT imaging. Nevertheless, with the availability of two CT machines since year 2020, many agreed that the process of getting a CT imaging has been sped up. The limited availability of MRI slots however, has made it difficult to extend the window therapy for thrombolysis. As described by one doctor (ID 01): “*we only thrombolyze those with(in) four and half hours. The guidelines now allow thrombolysis up to 9 h and if those patients with wake-up stroke, even up to 12 h which we do not have access to because that requires advanced imaging.”* Another doctor explained (ID 07): “*there is only one MRI machine and the queue is extremely long for MRI (even) for normal standard appointments”*.

##### Limited beds and space

The lack of beds and space has made it difficult to conduct thrombolysis and to accommodate another imaging machine in ED. As mentioned by one doctor (ID 11): “*Our emergency department layout and the space are very limited. There's no more space to expand”*.

##### Lack of human resources

There was a unanimous agreement that lack of manpower remained as an existing issue. One doctor said (ID 09): “*Not enough manpower, very busy. You need to run a ward, you need to do rounds, you need to do discharges, you need to attend clinics and then suddenly (when) thrombolysis calls, you have to go and attend.”* Echoed by another who shared how remote consultations due to shortage of neurologists occasionally led to delays (ID 07): “*When you were given images through WhatsApp, depending on your line, the quality of the video that is being sent, sometimes we do miss things. Sometimes it's very difficult to make the judgement call”*.

Heavy workload has prevented the conduct of thrombolysis in ED: “*We cannot spend the time to help you to monitor the patient. You can have either three CPR patient coming in 5 min apart or you can have polytrauma coming in* (ID 10).” Similarly, thrombolysis could not be initiated in CT suite for the same reason: “*our CT (functions) 24/7, so they (radiographers) cannot afford to let us dilute the medicine and jab there (CT suite)* (ID 01)”.

Furthermore, high turnover among nurses and medical officers was another issue brought up by one doctor (ID 12): “*The problem is training our nurses. Nurses that are specific for acute stroke care. Those are kind of hard to develop (but) once they are promoted, they might be transferred to another place. And then we have to train new nurses again”*.

##### Financial support for drug availability

Despite limited budget allocation, support from the higher authorities and other departments have been crucial in maintaining the availability of r-TPA. Said one doctor (ID 01): “*every time we said we needed it, they've (higher authorities) never said “No.” Our usage exceeded many times off budget.”* Proper budget planning has also been quoted as one substantial factor to receive enough funding to maintain the therapy: “*our pharmacist is doing a very good job in estimating all these (budget for r-TPA)* (ID 12)”.

## Discussion

The European Stroke Organization (ESO) aims to have at least 20% of all ischemic stroke patients being treated with thrombolysis by 2020 (18). Malaysia fared worse at nationwide than many other countries, at 1.6% among the public hospitals providing the therapy in 2018 (unpublished data: Hiew FL. Stroke Thrombolysis Survey in Ministry of Health Malaysia. 2019) although the rate in Hospital Z was higher at 20.8% in the same year, achieving the benchmarking rate set by ESO. A survey across 44 European countries reported an average of 7.3% of thrombolysis between 2016 and 2017 whereas country-specific rates in Europe were higher in Czech Republic (23.5% in 2018) and the Netherlands (21.7% in 2016) (Table 3) (3). The reported rates however, were noticeably lower in low and middle-income countries such as Thailand (7.8% in 2019) (19), Vietnam (5.6–8.5% in 2020) (20) and China (5.6% between 2019 and 2020) (21). Direct country comparison of the rates however, was not feasible owing to differences in study methods and reporting years.

**Table 3:** Comparison for rate of intravenous stroke thrombolysis by countries^a^.

**Country**	**Year**	**Rate of intravenous stroke** **thrombolysis (%)**
Malaysia	2013	5.2
	2019	15.1
Europe (3)	2016–2017	7.3
United States (2)	2018	13.7
Thailand (19)	2019	7.8
Vietnam (20)	2020	5.6–8.5
China (21)	2019–2020	5.6

^a^ Country-specific rates except for Malaysia (rates retrieved from a single hospital Z) and Europe (average estimate among 44 European countries).

In year 2019, Hospital Z has the highest uptake of intravenous stroke thrombolysis amongst other Ministry of Health hospitals in Malaysia. This is intriguing, considering that allocation of resources and hospital policies should be similar across all Ministry of Health hospitals. Geographically, the setting where this hospital is located remains mainly rural with poor access to healthcare services due to logistic difficulties (11). Findings from our case study has clearly observed two main factors facilitating the uptake in this therapy: (1) cohesiveness of team members, especially having positive interprofessional team dynamics and (2) facilitative work process. Patient factors were found to impede the uptake of thrombolysis, where almost two third of ischemic stroke patients arrived at the hospital outside the therapeutic window time, attributing patients' delayed presentation as a main barrier to the uptake of thrombolysis. Similarly, availability of resources was a barrier, although this was innovatively optimized to minimize its impact on the rate of the therapy. Only 3.8% of patients missed the opportunity to receive thrombolysis due to potential in-hospital delays.

One major contributing aspect to cohesiveness among team members was having positive interprofessional team dynamics. The concept of effective communication and understanding of one's role towards teamwork are crucial components to establish an engaging interprofessional team (22). Physician-driven stroke care without adequate involvement of other ground staff has been reported to lead to concerns of marginalization and disconnectedness (8). Having joint ownership of responsibility was another key facilitator. There have been global discussions surrounding the role of other doctors to provide thrombolysis, in particular the emergency and internal medicine physicians. Studies comparing neurologists and non-neurologist doctors on patients' functional outcomes and safety following the provision of intravenous stroke thrombolysis reported no differences between the groups (23, 24). In response to that, institutions in many different countries are adopting this approach to cope with shortages of neurologists; Hospital Z being one of the few in Malaysia.

Besides healthcare providers' belief and values attributing positively toward the therapy, leadership from the aspect of having quality stroke champions and support from higher authorities has also enabled optimization of available manpower and resources. These facilitators were consistent with findings from other studies; thus, exerting their importance in the uptake of thrombolysis (7, 25).

We also found that facilitative work processes have positively influenced the uptake of thrombolysis. Wang et al. reported how streamlining of workflow reduced in-hospital time delays for endovascular mechanical thrombectomy (26). A simplified pathway to increase the access to CT imaging for acute stroke interventions was also highlighted in the United Kingdom (27). Workflow simplification was quoted to bring about familiarity with one's roles. Understanding respective roles in the work process and that of other team members subsequently would give rise to a routine and coordinated stroke management (25). Furthermore, although the sense of urgency for rapid triage and assessment has often been associated with regular use of a written protocol for thrombolysis (8, 25), this factor was attributed to repetitive hands-on exposures to handle patients for the therapy in Hospital Z. Stecksen et al. echoed this, where lack of knowledge and experience was cited as a barrier to the implementation of stroke thrombolysis guidelines (7).

Consistently, patients' delayed presentation was a main barrier to hyperacute stroke care in Ghana (8). Likewise, 60.5% of Thai patients with ischemic stroke arrived late in the hospital (28) whereas in Lebanon, at 55.2% (29). This delay has been attributed to multiple reasons including but not limited to poor recognition of stroke symptoms, lack of awareness of the availability of a time-dependent therapy as well as poor accessibility (30).

Resource constraint is an issue of priority because a limited budget is almost always present. Results from studies conducted in high-income countries were parallel to our findings where restriction of resources could range from access to imaging facilities, beds, and space for an acute stroke unit, staff capacity, and finances (5, 7). The only difference could be the weightage that these factors carry to influence the uptake of thrombolysis. In a low- and middle-income country like ours, limited imaging facilities and staff especially neurologists are major impediments (8). Adding this limitation with heavy workload and high staff turnover, driving thrombolysis forward is often a major task.

### Strengths and weaknesses

The strength of our study lies in the efforts of applying the TICD framework at the initial stage to understand the components for implementation change in clinical practice but subsequently conducting an inductive analysis to derive explanations for the success of the therapy in Hospital Z and how the factors relate to each other, as shown in Figure 2. Furthermore, having quantitative data to triangulate with the qualitative findings not only adds depth to our current analysis but also functions as a strategy to strengthen the validity by connecting information from multiple data sources (31). Independent and cross coding theme derivation and quality checks were also conducted to minimize interpretation bias among researchers. This study described data from a single center. Comparable factors to that in the literature suggests potential generalizability of these findings. We acknowledge the possibility of selection bias as a result of the recruitment strategy but given that the interviews did not result in entirely positive points about the hospital, this bias should be minimal. Hierarchically, recruitment could not have been done without the permission from the Heads of Department.

### Implications on research and clinical practice

While acknowledging the ongoing constraints of resources as well as a lack of patients' awareness, what stood out as a lesson learnt was that the success in the uptake of thrombolysis in this hospital was attributed to cohesiveness of team members and having a facilitative work process. Understanding these facilitators which are modifiable within the service provision carry important implications for recommendations of targeted interventions to improve the uptake of the therapy in institutions of similar settings.

First, the theory of opportunistic dialogue where dialogues between team members are problem oriented, largely unplanned and facilitated by co-locations of team members and their commitment to work together can be applied to achieve a cohesive interprofessional team engagement (32). Moving away from the traditional multidisciplinary approach to adopt the concept of interdisciplinary team is pivotal to cultivate ownerships for responsibilities. Multidisciplinary refers to having knowledge from multiple disciplines brought together but each discipline acts from their own perspective within the boundaries of respective discipline. Interdisciplinary on the other hand, is defined as linking and integrating knowledge from different disciplines into one, using a coherent and coordinated approach. In other words, responsibilities are divided between disciplines in a multidisciplinary team. Contrastingly, responsibilities are shared among the different disciplines with interdisciplinary approaches, which provide an excellent learning and working environment where providers from other disciplines are able to learn and conduct tasks that are traditionally the roles of certain disciplines (33). In essence, interdependence is well-understood and acknowledged among team members in order to improve patient care. Interventions related to behavioral change have been advocated to promote such changes in team dynamics. These interventions were reported to have an increase of two times the odds of thrombolysis rate as compared to usual practice (9).

Second, lean techniques are increasingly used to streamline healthcare work processes. Value Stream Mapping (VSM) for example, has been applied to identify inefficiencies and to create a streamlined workflow to expedite time-dependent stroke care (34). Third, innovative education such as simulation workshops or mock stroke codes to provide hands-on experiences and opportunities to practice are foreseen to enhance familiarity of roles and instill the sense of urgency among healthcare providers. These strategies have been observed to improve the rate of thrombolysis (35, 36).

Fourth, research is an integral part of these recommendations. In the plans to implement any targeted interventions, it is vital to concurrently plan for an evaluation study to look at effectiveness and feasibility and importantly, the sustainability of such interventions. Several trials have been conducted for this purpose, with differing results (35–38).

## Conclusions

In conclusion, factors influencing the uptake of intravenous stroke thrombolysis have been identified from multiple aspects. Insight onto these factors is crucial to allow the development of targeted interventions to improve the provision of the therapy in countries of similar settings.

## Data availability statement

The datasets presented in this article are not readily available because of ethical and legal restrictions but is available from the corresponding author on reasonable request. Requests to access the datasets should be directed to WYH amyhwong@crc.gov.my.

## Ethics statement

The studies involving human participants were reviewed and approved by Medical Research and Ethics Committee, Ministry of Health Malaysia (NMRR ID: 19-3145-51552). The participants provided both their written and verbal informed consent to be interviewed in this study.

## Author contributions

WYH: conceptualization, methodology, investigation, formal analysis, and writing-original draft. SWN: investigation, formal analysis, and writing-original draft. SFT: methodology, investigation, formal analysis, and writing-review and editing. NAR: methodology, investigation, and writing-review and editing. WCL: data acquisition, formal analysis, and writing-review and editing. ZK, SKW, and SDP: data acquisition and writing-review and editing. SS: methodology and writing-review and editing. All authors contributed to the article and approved the submitted version.

## Funding

This study is funded by the Ministry of Health Malaysia research grant under the project Access to Hyperacute Stroke Care in Malaysia (NMRR 19-3145-51552). Ministry of Health Malaysia had no role in study design, data collection and analysis, decision to publish, or preparation of the manuscript.

## Conflict of interest

The authors declare that the research was conducted in the absence of any commercial or financial relationships that could be construed as a potential conflict of interest.

## Publisher's note

All claims expressed in this article are solely those of the authors and do not necessarily represent those of their affiliated organizations, or those of the publisher, the editors and the reviewers. Any product that may be evaluated in this article, or claim that may be made by its manufacturer, is not guaranteed or endorsed by the publisher.

## Abbreviations

TICD: tailored implementation of chronic diseases
MA: medical assistant/assistant medical officer
r-TPA: recombinant tissue plasminogen activator
IDI: In-depth interview
FGD: focus group discussion
ED: emergency department
CT: computed tomography
MRI: magnetic resonance imaging
ESO: European stroke organization
VSM: value stream mapping
TIA: transient ischemic attack.
