# Supplementary material for: Stroke thrombolysis in a middle-income country: A case study exploring the determinants of its implementation
Source: Front Neurol. 2022 Nov 24;13:1048807. doi: 10.3389/fneur.2022.1048807 (PMC9729841; doi:10.3389/fneur.2022.1048807)
Supplement: Supplementary file 2 [file Table_2.DOCX]

Supplementary Table 2. Factors influencing the uptake of intravenous stroke thrombolysis in Hospital Z.

| **No** | **Domain** | **Construct** | **Phase** | **Facilitator/Barrier** | **Codes** |
| --- | --- | --- | --- | --- | --- |
| 1 | Cohesiveness of team members | Positive interprofessional team dynamics: effective engagement among team members | Initial | Facilitator | availability of interdisciplinary meetings |
|  |  |  |  |  | initiative by neurologist to build rapport with other departments |
|  |  |  |  |  | effective communication to initiate and smoothen thrombolysis service |
|  |  |  | Current | Facilitator | opportunities for two way discussions with neurologists |
|  |  |  |  |  | willingness of neurosurgical team to cover stroke patients from district hospitals |
|  |  |  |  |  | awareness of thrombolysis service improved referrals from district hospitals |
|  |  |  |  |  | direct communication to request MRI by physicians in charge |
|  |  |  |  |  | effective intradepartment and interdepartment communication leads to agreement to prioritize stroke and resolves issues on bed reservation |
|  |  |  |  |  | trust in neurologists to make decisions for thrombolysis |
|  |  |  |  |  | sharing the task to push patients to CT scan |
|  |  |  |  |  | commitment from all departments |
|  |  |  |  |  | interdepartment teamwork smoothen thrombolysis |
|  |  |  |  |  | intradepartment teamwork allows new staff to learn on the job and responsibility to be shared |
|  |  |  |  |  | practice of teamwork within stroke team |
|  |  |  |  |  | teamwork spirit with accommodating radiologists |
|  |  |  |  |  | approachable leaders in various departments |
|  |  |  |  |  | positive attitude and liniency in guiding ground staff |
|  |  |  |  |  | positive work culture in keeping to standard of care |
|  |  |  |  | Barrier | interdepartment communication issues during referral |
|  |  | Positive interprofessional team dynamics: joint ownership of responsibility | Current | Facilitator | trust between healthcare providers of different professions |
|  |  |  |  |  | willingness to share responsibility in thrombolysis: to interpret CT image, to explain consent and to handle thrombolysis process |
|  |  |  |  |  | physicians from district hospitals sharing responsibility to thrombolyse patients |
|  |  |  |  |  | sharing responsibility to thrombolyse patients |
|  |  |  |  |  | positive attitude on workload with stroke thrombolysis service |
|  |  |  |  |  | positive attitude towards providing the service without any incentives |
|  |  |  |  |  | proactiveness to establish stroke thrombolysis training |
|  |  |  |  |  | priviledging non neurologist like physicians for thrombolysis |
|  |  |  |  |  | involvement of medical officers in thrombolysis to overcome lack of neurologists |
|  |  |  |  |  | systematic rotation for nurses to support stroke team |
|  |  |  |  |  | service extension to 24 hours |
|  |  |  |  | Barrier | different perspectives between healthcare providers on responsibility sharing to decide for thrombolysis (reluctance due to non familiarity, urgency of the service and existence of subspecialty) |
|  |  |  |  |  | lack of MOH policy on responsibility sharing among non neurologists for thrombolysis |
|  |  | Positive interprofessional team dynamics: non-threatening feedback | Current | Facilitator | availability and acceptance of constructive negative feedback |
|  |  |  |  |  | regular feedback to higher authorities on the outcome of service |
|  |  |  |  |  | feedback motivates staff and improve service |
|  |  |  |  |  | transfer of feedback to ground staff |
|  |  |  |  | Barrier | no regular feedback; available only upon issues |
|  |  |  |  |  | lack of regular audit to resolve issues |
|  |  | Shared personal beliefs and values | Current | Facilitator | intention and motivation to optimize the service |
|  |  |  |  |  | personal belief and experience on desired outcomes following thrombolysis |
|  |  |  |  |  | personal belief that benefits of thrombolysis is dependent on patients' circumstances |
|  |  |  |  |  | personal belief that thrombolysis is evidence-based |
|  |  |  |  |  | personal belief on workflow simplication |
|  |  |  |  |  | experience on good outcomes motivates healthcare providers |
|  |  |  |  |  | positive attitude toward expected outcome |
|  |  |  |  | Barrier | negative attitude toward expected outcome |
|  |  |  |  |  | wary on the benefits of thrombolysis |
|  |  | Passionate leadership: quality leadership in stroke champions | Current | Facilitator | willingness to be consulted directly for stroke cases |
|  |  |  |  |  | improves facilities for thrombolysis service |
|  |  |  |  |  | promotes awareness and training in stroke education |
|  |  |  |  |  | enthusiastic stroke champion motivates other departments |
|  |  |  |  |  | sharing of thrombolysis achievement motivates ground staff and other departments |
|  |  |  |  |  | positive attitude sets an example for medical officers |
|  |  |  |  |  | being hands-on promotes confidence among junior staff |
|  |  |  |  |  | use of KPIs to monitor and improve thrombolysis performance |
|  |  | Passionate leadership: institutional support | Current | Facilitator | appreciation from Hospital Director towards staff |
|  |  |  |  |  | initiative by MOH to improve the availability of imaging facilities |
|  |  |  |  |  | optimizing limited resources to support thrombolysis |
|  |  |  |  |  | initiative by Hospital Director to visualise data to improve thrombolysis |
|  |  |  |  |  | personal belief of Hospital Director to strive for better service |
|  |  |  |  |  | financial support from MOH for services with important outcomes |
|  |  |  |  |  | online training for thrombolysis by local stroke council |
|  |  |  |  |  | intention to set KPIs to improve door to needle time |
| 2 | Facilitative work process | Simplification of workflow | Initial | Barrier | (lack of) experience to handle thrombolysis |
|  |  |  | Current | Facilitator | workflow simplification to fasten thrombolysis process |
|  |  |  |  |  | improvement of patient flow in ED with stroke thrombolysis |
|  |  |  |  |  | establishment of standardised and systematic workflow |
|  |  |  |  |  | protocolised identification of stroke cases makes it straightforward |
|  |  |  |  |  | change of thrombolysis from ED to ward |
|  |  |  |  |  | liniency in setting up criteria to identify potential stroke cases to avoid missed diagnosis |
|  |  |  |  |  | intention to simplify thrombolysis workflow |
|  |  |  |  |  | intention to optimise pre-hospital workflow for stroke |
|  |  |  |  |  | availability of standardized documents to aid hospital's acute stroke pathway: pre-hospital and in-hospital stroke protocol, thrombolysis stamp, use of technology) |
|  |  |  |  |  | various innovation by radiology department to improve the time needed to provide CT images |
|  |  |  |  |  | competent ground staff |
|  |  |  |  |  | familiarity with workflow and roles among staff |
|  |  |  |  |  | competent and confident to interpret CT images |
|  |  |  |  | Barrier | difficult to identify stroke patients due to atypical symptoms or concurrent presenting complaints |
|  |  | Understanding the rationale for the sense of urgency | Initial | Barrier | lack of sense of urgency among ED staff |
|  |  |  |  |  | pressure from the urgency of the timing affect the service |
|  |  |  | Current | Facilitator | awareness of the urgency to identify stroke patients |
|  |  |  |  |  | sense of urgency among ground staff and emergency physician |
|  |  |  |  |  | rapid response from stroke/medical team for stroke activation |
|  |  |  |  |  | establishment of stroke thrombolysis training |
|  |  |  |  |  | awareness of the need for training |
|  |  |  |  |  | gaining experience from working with stroke team |
|  |  |  |  |  | working experience improves identification of stroke patients |
|  |  |  |  |  | practice improves stroke examination |
|  |  |  |  |  | establishment of stroke thrombolysis training for PPWs |
|  |  |  |  | Barrier | lack of structured training among ED nurses |
|  |  | Guidelines | Current | Facilitator | sufficient guidelines available |
|  |  |  |  | Barrier | unclear guidelines on allowable BP level for thrombolysis |
|  |  |  |  |  | outdated guidelines for thrombolysis in 2012 |
| 3 | Patient factors | Delayed presentation | Current | Facilitator | contradicting views on public awareness of stroke symptoms |
|  |  |  |  | Barrier | logistic issues |
|  |  |  |  |  | awareness of stroke symptoms among young patients |
|  |  |  |  |  | presentation to hospital dependent on symptom severity |
|  |  | Patient comorbidities | Current | Barrier | poor patient condition makes it challenging to identify stroke cases at triage |
|  |  |  |  |  | patient comorbidities prevents the uptake of thrombolysis |
|  |  |  |  |  | unstable patients causing delay to CT scan |
|  |  | Consent of therapy | Current | Facilitator | patient consent dependent on doctor-patient communication |
|  |  |  |  | Barrier | delay and influence by family members |
|  |  |  |  |  | language barrier |
|  |  | Unclear stroke-related history | Current | Barrier | unclear clinical history |
|  |  |  |  |  | uncooperative caller/relatives during pre-hospital care assessment |
|  |  |  |  |  | language barrier |
| 7 | Availability of resources | Resource-intensive therapy | Initial | Barrier | detailed requirements for thrombolysis |
|  |  |  |  |  | resource intensive requirements for thrombolysis |
|  |  |  |  |  | risk of bleeding with thrombolysis |
|  |  |  | Current | Facilitator | short duration of CT imaging process for thrombolysis |
|  |  | Limited imaging facilities | Current | Facilitator | availability of 2 CT machines |
|  |  |  |  | Barrier | lack of CT imaging in ED |
|  |  |  |  |  | lack of CT imaging in district hospitals |
|  |  |  |  |  | CT room far from stroke unit and ED |
|  |  |  |  |  | lack of MRI/CT perfusion machine |
|  |  | Limited space and beds | Current | Facilitator | availability of a weighing bed |
|  |  |  |  |  | availability of an acute stroke unit |
|  |  |  |  | Barrier | lack of beds and space in ED |
|  |  |  |  |  | lack of space in hospital to accommodate another MRI |
|  |  |  |  |  | lack of space and staff in CT suite to allow for thrombolysis |
|  |  | Lack of human resources | Current | Facilitator | availability of a stroke team |
|  |  |  |  |  | availability of ED pharmacists |
|  |  |  |  |  | availability of support staff after office hours |
|  |  |  |  |  | availability of stroke nurses |
|  |  |  |  |  | support from higher authorities to provide manpower |
|  |  |  |  | Barrier | high turnover among medical MOs and nurses |
|  |  |  |  |  | lack of manpower during change of shift in ED |
|  |  |  |  |  | remote consultation after office hours resulting in delays in deciding for thrombolysis |
|  |  |  |  |  | lack of neurologists |
|  |  |  |  |  | lack of radiographers for MRI service after office hours |
|  |  |  |  |  | lack of manpower in ED: staff to monitor post thrombolysis, porter and onsite pharmacist after office hours |
|  |  |  |  |  | heavy workload/work burden, especially in ED and CT room |
|  |  | Financial support for drug availability | Current | Facilitator | support from other departments to maintain drug availability |
|  |  |  |  |  | availability of the service improves the budget |
|  |  |  |  |  | proper drug budget planning |
|  |  |  |  |  | one-off usage of drug for thrombolysis makes it a priority |
|  |  |  |  |  | willingness to supply drugs to other hospitals |
|  |  |  |  | Barrier | lack of neurology's own budget |
|  |  | Availability of data | Current | Facilitator | availability of stroke data to support thrombolysis service |

*Grayed rows refer to barriers whereas non-grayed rows refer to facilitators
